# Supplementary material for: Dogs tuned to conspecific vocalizations: behavioral evidence for a voice processing preference
Source: Anim Cogn. 2026 Jun 19;29(1):62. doi: 10.1007/s10071-026-02074-0 (PMC13529616; doi:10.1007/s10071-026-02074-0)
Supplement: Supplementary file 1 [file 10071_2026_2074_MOESM1_ESM.pdf]

Animal Cognition

Dogs tuned to conspecific vocalizations: behavioral evidence for a voice processing preference

Tamás Faragó[1,2], Kinga Surányi[1,3,4,5,*], Flavie Bensaali-Nemes[1,3], Elodie Ferrando[1], Zsófia Szatlószki [1,3], Attila Andics[1,3,6]

[1]Department of Ethology, ELTE Eötvös Loránd University, Budapest, Hungary

[2]BARKS Lab, Department of Ethology, Eötvös Loránd University, Budapest, Hungary

[3]Neuroethology of Communication Lab, Department of Ethology, ELTE Eötvös Loránd University, Budapest, Hungary

[4]Doctoral School of Biology, Institute of Biology, ELTE Eötvös Loránd University, Budapest, Hungary

[5]Hungarian National Museum Public Collection Centre – Hungarian Natural History Museum, Department of Zoology, Budapest, Hungary

[6]ELTE NAP Canine Brain Research Group, Budapest, Hungary

*Corresponding author. E-mail address: kingablanka08@gmail.com (K. Surányi)

Supplementary Table

Supplementary Table S1 Subjects of the study

ID	Name	Breed	Sex	Age
1	Bodza	Mixed breed	Female	4
2	Biju	Siberian husky	Female	7.5
3	Bogyó	Mixed breed	Female	11.5
4	Pityke	West highland white terrier	Male	8
5	Gesztenye	Mixed breed	Female	3.5
6	Menta	Mixed breed	Female	6
7	Millie	Mixed breed	Female	3
8	Mambo	Mixed breed	Female	2.5
9	Ben	Australian kelpie	Male	3
10	Csele	Mudi	Female	11
11	Joel	Mixed breed	Male	3
12	Ábránd	Mixed breed	Male	5
13	Berni	English cocker spaniel	Male	7
14	Mira	Golden retriever	Female	8.5
15	Nara	Belgian Malinois	Female	6
16	Kali	Dutch shepherd	Female	1
17	Károly	Hungarian vizsla	Male	6

18	Cody	Mixed breed	Male	1.5
19	Zsebi	Mixed breed	Female	2
20	Netti	English cocker spaniel	Female	4
21	Milla	Miniature poodle	Female	2
22	Lia	English cocker spaniel	Female	4
23	Fondue	Mixed breed	Female	2.5
24	Vilmos	Pug	Male	9
25	Rozi	Hungarian vizsla	Female	5.5
26	Cherry	German shepherd (Alsatian)	Female	4
27	Indie	Belgian Tervuren	Female	3
28	Zsömi	Maltese	Female	9
29	Szaffi	English cocker spaniel	Female	5
30	Kumisz	Mixed breed	Male	12
31	Nala	Mixed breed	Female	3
32	Pedro	Dachshund (wire haired)	Male	1
33	Luna	Bull terrier	Female	11
34	Vilma	Bedlington terrier	Female	2.5
35	Bogi	Lagotto Romagnolo	Female	5
36	Piszke	Pumi	Male	5
37	Réka	Mixed breed	Female	7
38	Charlie	Czechoslovakian wolfdog	Male	10
39	Aria	Fox terrier (smooth)	Female	1
40	Tito	Mixed breed	Male	8

This table shows the identification number (ID), name, breed, sex, and age of the dogs who participated in the study (and were included in the statistical analysis)
